# Disparities in Learning Mode Access Among K–12 Students During the COVID-19 Pandemic, by Race/Ethnicity, Geography, and Grade Level — United States, September 2020–April 2021

**DOI:** 10.15585/mmwr.mm7026e2

**Published:** 2021-07-02

**Authors:** Emily Oster, Rebecca Jack, Clare Halloran, John Schoof, Diana McLeod, Haisheng Yang, Julie Roche, Dennis Roche

**Affiliations:** ^1^COVID-19 School Response Dashboard, Providence, Rhode Island; ^2^Brown University, Providence, Rhode Island; ^3^Abt Associates, Chamblee, Georgia; ^4^Burbio, New York, New York; ^5^Precision Development, Boston, Massachusetts.

In response to the COVID-19 pandemic, schools across the United States began transitioning to virtual learning during spring 2020. However, schools’ learning modes varied during the 2020–21 school year across states as schools transitioned at differing times back to in-person learning, in part reflecting updated CDC guidance. Reduced access to in-person learning is associated with poorer learning outcomes and adverse mental health and behavioral effects in children (*1*–*3*). Data on the learning modes available in 1,200 U.S. public school districts (representing 46% of kindergarten through grade 12 [K–12] public school enrollment) from all 50 states and the District of Columbia during September 2020–April 2021 were matched with National Center for Education Statistics (NCES) demographic data. Learning mode access was assessed for K–12 students during the COVID-19 pandemic, over time and by student race/ethnicity, geography, and grade level group. Across all assessed racial/ethnic groups, prevalence of virtual-only learning showed more variability during September–December 2020 but declined steadily from January to April 2021. During January–April 2021, access to full-time in-person learning for non-Hispanic White students increased by 36.6 percentage points (from 38.0% to 74.6%), compared with 31.1 percentage points for non-Hispanic Black students (from 32.3% to 63.4%), 23.0 percentage points for Hispanic students (from 35.9% to 58.9%) and 30.6 percentage points for students of other races/ethnicities (from 26.3% to 56.9%). In January 2021, 39% of students in grades K–5 had access to full-time in-person learning compared with 33% of students in grades 6–8 and 30% of students in grades 9–12. Disparities in full-time in-person learning by race/ethnicity existed across school levels and by geographic region and state. These disparities underscore the importance of prioritizing equitable access to this learning mode for the 2021–22 school year. To increase equitable access to full-time in-person learning for the 2021–22 school year, school leaders should focus on providing safety-optimized in-person learning options across grade levels. CDC’s K–12 operational strategy presents a pathway for schools to safely provide in-person learning through implementing recommended prevention strategies, increasing vaccination rates for teachers and older students with a focus on vaccine equity, and reducing community transmission (*4*).

All data for the analyses were publicly available. Data were collected on learning modes used across 1,200 school districts from all 50 states and the District of Columbia, representing 46% of U.S. K–12 public school enrollment and 90% of students in the 232 most populous U.S. counties.* Information on learning mode was collected through weekly Internet searches of school district webpages, Facebook, and other public sources for each school district, by grade level group (K–5, 6–8, 9–12) or individual grade level, as available, and were classified using the most in-person mode available.^†^ Learning modes were categorized as “full-time in-person” (i.e., access to in-person learning 5 days a week), “virtual-only” (i.e., no access to in-person learning; entirely online, synchronous and asynchronous), or “hybrid” (i.e., access to part-time in-person learning). Data were collected weekly during January–April 2021 and less frequently during September–December 2020 because data collection was not systematized until December 2020.

District enrollment data from the 2019–20 NCES Common Core of Data collected by the U.S. Department of Education (*5*) were used to estimate enrollment in each of the 1,200 assessed school districts. District and grade-level enrollment data by race/ethnicity from the NCES data were matched to learning mode data to estimate weekly numbers of students with access to each learning mode, by race/ethnicity, geography (state and region), and grade level group. The analytic time frame was September 8, 2020–April 23, 2021. Weekly variation in school learning mode was examined over the 2020–21 school year by race/ethnicity for non-Hispanic White students, non-Hispanic Black students, Hispanic students (of any race), and students of other races/ethnicities^§^; weekly variation was also assessed by grade level for non-Hispanic White students and students of color.^¶^ To analyze differences in access to virtual-only, hybrid, and full-time in-person learning modes between non-Hispanic White students and students of color by region** and state, CDC calculated the mean share of access^††^ to learning modes over the entire study period. Trends over time for each race/ethnicity group were analyzed using linear regressions of percentage of students with access on number of weeks from the start of the study period with total district enrollment for the race/ethnicity group as analytic weights. To compare regions and states, the mean percentage of students with access and 95% confidence intervals for the entire study period were calculated using total district enrollment as analytic weights. Stata software (version 16.0; StataCorp) was used to conduct all analyses. This activity was reviewed by CDC and was conducted consistent with applicable federal law and CDC policy.^§§^

Full-time in-person learning access steadily increased starting January 2021 among all assessed racial/ethnic groups (p< 0.01) (Figure 1). During January–April 2021, access to full-time in-person learning for non-Hispanic White students increased by 36.6 percentage points (from 38.0% to 74.6%) compared with 31.1 percentage points for non-Hispanic Black students (from 32.3% to 63.4%), 23.0 percentage points for Hispanic students (from 35.9% to 58.9%), and 30.6 percentage points for students of other races/ethnicities (from 26.3% to 56.9%) (Figure 1). Access to hybrid learning increased by 9.5 percentage points for non-Hispanic White students (from 13.9% to 23.4%) compared with 21.7 percentage points for non-Hispanic Black students (from 8.3% to 30.0%), 23 percentage points for Hispanic students (from 9.7% to 32.7%), and 24.6 percentage points for students of other races/ethnicities (from 12.3% to 36.9%) (Figure 1). Across all assessed racial/ethnic groups, prevalence of virtual-only learning decreased significantly during September 2020–April 2021 (Figure 1).

**FIGURE 1:**
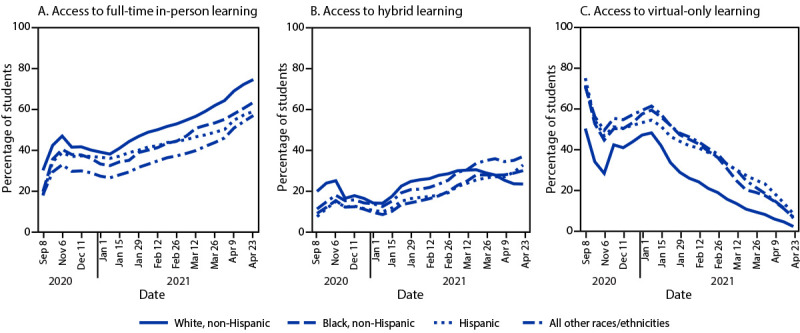
Changes in access to full-time in-person (A), hybrid (B), and virtual-only (C) learning,* by race/ethnicity^†^ — United States, September 2020–April 2021^§^^,^^¶^ * Learning modes are defined as “full-time in-person” (access to in-person learning 5 days a week), “hybrid” (access to part-time in-person learning), and “virtual-only” (no access to in-person learning; entirely online). ^†^ Race/ethnicity data are based on district-level National Center for Education Statistics 2019–20 demographic data (https://nces.ed.gov/ccd/elsi). Hispanic students could be of any race. Students included in “All other races/ethnicities” include non-Hispanic students who are American Indian or Alaska Native, Asian or Pacific Islander, or two or more races. ^§^ Data before January 1, 2021, were collected less frequently and are not presented at weekly intervals. Data during January 1–April 23, 2021, are presented on a weekly basis. Date labels are condensed for readability. ^¶^ Access to full-time in-person learning increased significantly for all races/ethnicities (p<0.01 for all four regressions), access to hybrid learning increased significantly for all races/ethnicities (p<0.01 for all four regressions), and access to virtual learning decreased significantly for all races/ethnicities (p<0.01 for all four regressions).

During January–April 2021, the percentage of students with access to virtual-only learning decreased by 46.0 percentage points for non-Hispanic White students (48.1% to 2.1%), 52.6 percentage points for non-Hispanic Black students (59.3% to 6.7%), 46.1 percentage points for Hispanic students (54.4% to 8.3%), and 55.2 percentage points for students of other races/ethnicities (61.3% to 6.1%). During September 2020-April 2021, students in the South had greater access to full-time in-person learning (62.5%), on average, compared with other regions (Midwest, 37.1%; Northeast, 16.2%; and West, 21.8%). Access to in-person learning varied by state with the lowest mean percent of all students with access in Hawaii (1.3%) and highest in Wyoming and Montana (100%) (Table). In 43 states, access to full-time in-person learning was higher for non-Hispanic White students compared with students of color. The District of Columbia, Delaware, Hawaii, Wyoming, and Montana had the lowest disparity; Ohio and Pennsylvania had the highest.

**TABLE:** Mean difference in access* to full-time in-person compared with virtual-only learning modes^†^ between non-Hispanic White students and students of color,^§^ by region and jurisdiction^¶^ — United States, September 2020–April 2021

Area	Total enrollment included in sample	Full-time in-person access		Virtual-only access		
		Mean percentage of students with access (95% CI)	Mean difference in access for students of color (95% CI)		Mean percentage of students with access (95% CI)	Mean difference in access for students of color (95% CI)
**Region**						
South	11,733,585	62.5 (61.4 to 63.5)	−3.5 (−4.5 to −2.5)		21.6 (20.7 to 22.5)	3.8 (2.2 to 5.5)
Midwest	3,280,369	37.1 (36.1 to 38.1)	−20.1 (−21.7 to −18.4)		36.7 (35.7 to 37.8)	22.6 (19.3 to 25.9)
West	5,451,104	21.8 (20.8 to 22.7)	−22.6 (−24.3 to −20.9)		58.4 (57.2 to 59.6)	26.7 (24.3 to 29.2)
Northeast	1,974,998	16.2 (15.5 to 17.0)	−12.3 (−14.8 to −9.9)		41.7 (40.6 to 42.8)	31.0 (28.8 to 33.2)
**Jurisdiction**						
Wyoming	27,751	100.0 (100.0)	0 (—)		0 (—)	0 (—)
Montana	12,488	100.0 (100.0)	0 (—)		0 (—)	0 (—)
Florida	2,679,579	98.4 (97.6 to 99.2)	−1.1 (−3.2 to 1.1)		1.3 (0.5 to 2.1)	1.1 (−1.1 to 3.3)
Arkansas	102,025	81.5 (75.5 to 87.5)	21.3 (20.6 to 22.0)		1.0 (−0.5 to 2.5)	0.4 (−0.4 to 1.1)
Utah	435,494	79.5 (74.7 to 84.3)	−18.9 (−21.0 to −16.9)		2.7 (0.7 to 4.6)	3.6 (2.1 to 5.0)
South Dakota	43,311	76.8 (66.7 to 86.8)	−0.8 (−1.0 to −0.6)		0.0 (—)	0 (—)
Texas	3,054,742	74.8 (73.1 to 76.5)	−13.5 (−15.1 to −11.9)		5.8 (4.9 to 6.8)	4.3 (2.1 to 6.4)
Louisiana	257,164	74.6 (71.0 to 78.1)	−11.0 (−12.5 to −9.5)		1.2 (−0.3 to 2.6)	1.3 (−0.4 to 2.9)
Nebraska	146,720	73.6 (68.8 to 78.5)	−10.6 (−17.9 to −3.3)		3.8 (1.6 to 5.9)	3.8 (0.0 to 7.7)
Alabama	293,702	69.5 (64.3 to 74.6)	−8.8 (−13.2 to −4.5)		17.3 (13.1 to 21.5)	14.8 (10.4 to 19.1)
Mississippi	120,489	69.2 (63.4 to 75.0)	−16.3 (−22.0 to −10.7)		11.2 (6.9 to 15.4)	15.8 (9.1 to 22.5)
Georgia	1,012,693	68.5 (64.5 to 72.6)	−17.3 (−18.0 to −16.6)		23.9 (20.1 to 27.6)	15.1 (12.7 to 17.5)
South Carolina	497,693	67.7 (64.3 to 71.1)	−2.2 (−3.1 to −1.4)		8.9 (6.4 to 11.3)	2.8 (0.9 to 4.8)
North Dakota	44,341	65.8 (57.5 to 74.2)	0.1 (−1.1 to 1.3)		0.6 (−0.6 to 1.8)	0.1 (−0.1 to 0.3)
Arizona	348,120	64.7 (60.4 to 69.1)	−14.2 (−17.2 to −11.2)		25.6 (21.5 to 29.8)	15.6 (11.6 to 19.6)
Iowa	124,369	60.0 (53.8 to 66.2)	−7.1 (−10.3 to −3.8)		10.7 (6.9 to 14.6)	3.7 (0.7 to 6.7)
Tennessee	494,768	58.8 (53.2 to 64.3)	−16.9 (−24.7 to −9.1)		36.6 (30.9 to 42.2)	21.7 (13.2 to 30.1)
Missouri	271,026	55.8 (52.3 to 59.3)	−14.1 (−15.8 to −12.4)		21.5 (18.1 to 24.9)	22.8 (18.6 to 27.0)
Indiana	328,466	55.1 (52.4 to 57.8)	−14.7 (−16.1 to −13.4)		16.1 (13.7 to 18.6)	10.9 (7.1 to 14.8)
Oklahoma	153,078	53.7 (48.1 to 59.3)	−20.5 (−25.8 to −15.1)		26.7 (21.3 to 32.1)	18.1 (11.4 to 24.8)
Kansas	184,604	52.9 (48.1 to 57.7)	−7.4 (−10.9 to −4.0)		29.3 (23.9 to 34.7)	15.0 (11.6 to 18.3)
Idaho	126,946	44.8 (39.4 to 50.2)	−8.4 (−10.3 to −6.5)		13.2 (8.3 to 18.0)	5.0 (1.7 to 8.3)
Colorado	651,020	44.3 (41.5 to 47.2)	−4.6 (−6.2 to −3.0)		28.7 (25.0 to 32.4)	2.4 (0.0 to 4.9)
Vermont	11,215	44.1 (38.1 to 50.2)	−1.5 (−4.1 to 1.0)		8.5 (4.8 to 12.3)	4.4 (2.1 to 6.7)
Michigan	345,524	40.9 (38.5 to 43.2)	−20.7 (−26.8 to −14.7)		44.7 (42.2 to 47.2)	21.6 (15.8 to 27.4)
Alaska	70,370	40.1 (31.9 to 48.3)	−1.4 (−4.9 to 2.1)		41.6 (31.2 to 52.0)	12.5 (8.9 to 16.1)
West Virginia	56,868	39.9 (28.4 to 51.4)	−0.7 (−2.4 to 0.9)		28.4 (18.0 to 38.8)	1.2 (−0.7 to 3.0)
Ohio	499,577	36.8 (34.5 to 39.2)	−23.2 (−25.4 to −21.0)		32.1 (29.9 to 34.4)	21.8 (16.2 to 27.4)
Connecticut	143,101	35.4 (31.9 to 38.9)	−9.8 (−13.4 to −6.3)		19.1 (15.8 to 22.4)	9.9 (7.0 to 12.9)
Rhode Island	43,015	35.1 (30.9 to 39.3)	3.6 (0.8 to 6.4)		26.7 (19.6 to 33.8)	−3.3 (−8.4 to 1.8)
Minnesota	227,000	30.4 (26.5 to 34.3)	−2.1 (−3.6 to −0.5)		50.2 (45.4 to 55.0)	11.9 (8.6 to 15.1)
North Carolina	942,072	25.5 (23.0 to 28.0)	−4.6 (−5.4 to −3.7)		38.5 (34.7 to 42.2)	10.9 (7.9 to 13.8)
Wisconsin	268,237	25.5 (22.3 to 28.8)	−12.9 (−15.7 to −10.2)		59.6 (55.5 to 63.7)	27.3 (22.7 to 31.9)
Pennsylvania	633,775	22.4 (20.8 to 24.0)	−21.5 (−25.6 to −17.5)		44.1 (42.1 to 46.2)	38.6 (35.7 to 41.6)
Kentucky	199,713	17.8 (12.3 to 23.3)	−9.0 (−11.3 to −6.8)		63.4 (56.3 to 70.4)	12.6 (8.4 to 16.7)
Delaware	90,500	15.1 (11.7 to 18.6)	0.0 (−1.1 to 1.0)		27.1 (21.4 to 32.7)	4.1 (1.9 to 6.3)
New Mexico	170,693	14.9 (9.5 to 20.2)	−1.2 (−1.6 to −0.7)		77.2 (71.2 to 83.2)	3.2 (2.0 to 4.3)
New Hampshire	52,543	14.8 (10.7 to 18.9)	−8.5 (−11.4 to −5.5)		25.8 (20.7 to 30.8)	10.7 (6.3 to 15.1)
Nevada	408,723	13.6 (8.6 to 18.5)	−6.4 (−7.3 to −5.4)		65.7 (56.4 to 75.1)	10.8 (8.6 to 12.9)
New York	377,921	13.5 (12.3 to 14.8)	−5.7 (−7.0 to −4.4)		25.1 (23.1 to 27.1)	14.3 (10.9 to 17.7)
Virginia	873,746	12.2 (9.9 to 14.5)	−7.1 (−8.3 to −5.9)		59.2 (55.5 to 62.9)	8.0 (6.8 to 9.1)
Illinois	797,194	10.1 (8.7 to 11.6)	−9.7 (−13.2 to −6.3)		54.0 (51.5 to 56.5)	21.4 (16.7 to 26.1)
Maine	27,647	7.9 (4.6 to 11.3)	−3.1 (−4.8 to −1.5)		3.4 (1.0 to 5.8)	−1.7 (−4.0 to 0.5)
District of Columbia	50,971	7.0 (2.9 to 11.2)	0 (—)		89.6 (85.2 to 94.0)	0 (—)
Massachusetts	239,342	6.8 (5.2 to 8.3)	−4.6 (−8.0 to −1.1)		54.9 (51.2 to 58.5)	32.8 (28.2 to 37.3)
New Jersey	446,439	6.7 (5.5 to 7.9)	−8.5 (−12.5 to −4.4)		59.2 (56.7 to 61.7)	41.4 (37.4 to 45.4)
Oregon	302,998	4.4 (3.1 to 5.7)	−2.5 (−3.5 to −1.5)		80.5 (77.5 to 83.5)	5.5 (3.6 to 7.4)
California	2,327,278	4.0 (3.3 to 4.6)	−5.8 (−6.8 to −4.8)		79.1 (77.6 to 80.6)	17.4 (15.0 to 19.8)
Washington	388,135	2.8 (2.2 to 3.5)	−1.1 (−1.4 to −0.8)		69.0 (66.2 to 71.8)	5.6 (4.1 to 7.1)
Maryland	853,781	2.3 (0.9 to 3.8)	−3.5 (−6.1 to −0.9)		76.9 (73.0 to 80.8)	11.3 (6.4 to 16.1)
Hawaii	181,088	1.3 (−0.3 to 3.0)	0 (—)		52.3 (42.1 to 62.4)	0 (—)

* To calculate mean difference, the percentage of students with access to virtual-only and full-time in-person learning modes was first calculated for each time point during September 2020–April 2021. The average of these percentages was then calculated over the study period for each learning mode. The percentage point difference of these two means is presented. A positive value indicates a higher percentage of students of color in the learning mode compared with non-Hispanic White students. A negative value indicates a higher percentage of non-Hispanic White students in the learning mode compared with students of color.

^†^ The “virtual-only” learning mode is defined as no access to in-person instruction; entirely online, including synchronous and asynchronous instruction. The “full-time in-person” learning mode is defined as access to in-person instruction 5 days a week.

^§^ Race/ethnicity data are based on district-level National Center for Education Statistics 2019–20 demographic data (https://nces.ed.gov/ccd/elsi). Students of color include all students who identify with a race/ethnicity other than non-Hispanic White, including students who are American Indian or Alaska Native, Asian or Pacific Islander, Black or African American, Hispanic, or two or more races.

**^¶^** Sample includes students who had access to all learning modes, including virtual-only instruction, full-time in-person instruction, and hybrid (access to part-time in-person learning) instruction and mean percent of students with access and 95% confidence intervals are calculated using total district enrollment as analytic weights. Note that the percent of students with access to hybrid instruction is not presented in this table to highlight a focus on virtual access and full-time in-person access. Thus, the columns presenting access to virtual-only and full-time in-person instruction might not sum to 100%.

As of January 8, 39% of K–5 students had access to full-time in-person learning compared with 33% of students in grades 6–8 and 30% of students in grades 9–12; however, differences in full-time in-person learning by race/ethnicity were noted across elementary, middle, and high school levels. During January–April 2021, the difference in access to full-time in-person learning between non-Hispanic White students and students of color in grades K–5 increased by 6.9 percentage points (8.2 percentage points to 15.1 percentage points) compared with increases of 11.4 percentage points at the middle school level (from 2.4 to 13.8) and 12.7 percentage points at the high school level (from 2.1 to 14.8) (Figure 2).

**FIGURE 2:**
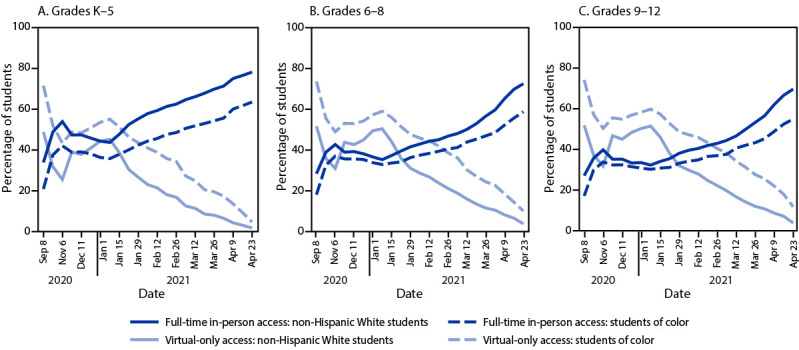
Student access to learning modes,* by grade level and race/ethnicity^†^ — United States, September 2020–April 2021^§^^,^^¶^ * Learning modes are defined as “full-time in-person” (access to in-person learning 5 days a week) and “virtual-only” (no access to in-person learning; entirely online). ^†^ Race/ethnicity data are based on district-level National Center for Education Statistics 2019–20 demographic data (https://nces.ed.gov/ccd/elsi). The “Students of color” category includes all students not identified as non-Hispanic White, including students who are American Indian or Alaska Native, Asian or Pacific Islander, Black or African American, Hispanic, or two or more races. ^§^ Data before January 1, 2021, were collected less frequently and are not presented at weekly intervals. Data during January 1–April 23, 2021, are presented on a weekly basis. Date labels are condensed for readability. ^¶^ Trends over time for non-Hispanic White students and students of color by grade level were analyzed using linear regressions of percentage of students with access on number of weeks from the start of the study period with the grade level group’s total district enrollment for the race/ethnicity group as analytic weights. Access to full-time in-person learning increased significantly for all three grade level groups for both non-Hispanic White students and students of color (p<0.01 for all four regressions), and access to virtual learning decreased significantly for all three grade level groups for both non-Hispanic White students and students of color (p<0.01 for all four regressions).

## Discussion

During January–April 2021, overall access to full-time in-person learning increased for all K–12 students. However, disparities in access to full-time in-person learning were apparent by race/ethnicity, geography, and school level. The populations with the most access to full-time in-person learning were non-Hispanic White students, students living in the South, and those in grades K–5. These disparities in learning mode during the COVID-19 pandemic underscore the importance of decreasing community transmission and of increasing equitable access to full-time in-person learning for the 2021–22 school year.

Growing evidence suggests virtual learning can be a challenge for many students, leading to learning losses for children and worsening mental health for children and parents (*1*–*3*). Therefore, disparities in access to full-time in-person learning across demographic groups might translate into short-term increases in educational disparities; however, such disparities might be driven by a number of factors (*1*). For example, urban districts might be less likely to open for full-time in-person learning, in part because of higher COVID-19 community rates, and these districts generally include more students of color (*6*). Further, rates of COVID-19 hospitalization and mortality have been higher in communities of color, and districts serving a larger share of these students might have faced more significant public health challenges as they made decisions about reopening schools (*7*,*8*).

The findings in this report are subject to at least five limitations. First, the study assessed access to different learning modes and not how students actually received instruction. Some evidence suggests that families of color are less likely to opt in to full-time in-person school, even when it is an option, because they are more likely to be concerned about their child contracting COVID-19 and about students not complying with COVID-19 mitigation practices in schools (*9*). Second, data included in this report cover only 1,200 school districts out of the 13,057 in the nation (*5*), representing only 46% of public K–12 enrollment in the United States; therefore, although the sampling frame is more representative of larger districts in more populated areas, it is not representative of the entire United States. Third, data were collected from public sources that could reflect inaccuracies if not updated frequently. Fourth, data were collected less frequently during September–December 2020 because data collection was not systematized until December 2020. Finally, these data do not directly measure changes in learning outcomes; such outcomes might be affected by types of learning modes (*1*).

This study documents disparate access to full-time in-person learning across racial/ethnic groups among U.S. K–12 students over the 2020–21 school year, by geography and school level. These results highlight the importance of continued efforts to address inequities in access to the full-time in-person learning mode, including increasing vaccination coverage to reduce community transmission in all populations. Evidence suggests that many K–12 schools that have optimized prevention strategies have safely opened for full-time in-person learning and remained open (*10*). To increase equitable access to full-time in-person learning for the 2021–22 school year, school leaders should focus on providing safety-optimized in-person learning options across grade levels. CDC’s K–12 operational strategy presents a pathway for schools to safely provide in-person learning through implementation of recommended prevention strategies, increasing vaccination rates, and reducing community transmission (*4*).

**Summary**

**What is already known about this topic?**

Reduced access to in-person learning is associated with poorer learning outcomes and adverse mental health and behavioral effects in children.

**What is added by the report?**

Although access to in-person, hybrid, and virtual learning modes varied throughout the school year, during January–April 2021, access to full-time in-person learning for non-Hispanic White students increased by 36.6 percentage points, 31.1 percentage points for non-Hispanic Black students, 22.0 percentage points for Hispanic students, and 26.6 percentage points for students of other race/ethnicities.

**What are the implications for public health practice?**

To increase equitable access to full-time in-person learning for the 2021–22 school year, school leaders should focus on providing safety-optimized in-person learning options across grade levels in all geographic areas. Vaccination and other efforts to reduce levels of community transmission should be intensified.

## Footnotes

*
https://about.burbio.com/methodology/

† For example, districts that offered both full-time in-person and hybrid options to K–5 students are categorized as “full-time in-person for K–5.” Grade levels categorized as virtual do not have access to hybrid or full-time in-person learning modes.

§ Other race/ethnicities includes students who identify as American Indian or Alaska Native, Asian or Pacific Islander, or two or more races.

¶ “Students of color” includes all students who identify with a race/ethnicity group other than non-Hispanic White.

** Regions of the United States are defined by NCES. https://nces.ed.gov/nationsreportcard/hsts/tabulations/regions.asp

†† To calculate mean difference, the percentage of students with access to virtual-only and full-time in-person learning modes was first calculated for each time point during September 2020–April 2021. The average of these percentages was then calculated over the study period for each learning mode. The percentage point difference of these two means is presented. A positive value indicates a higher percentage of students of color in the learning mode compared with non-Hispanic White students. A negative value indicates a higher percentage of non-Hispanic White students in the learning mode compared with students of color.

§§ 45 C.F.R. part 46, 21 C.F.R. part 56; 42 U.S.C. Sect. 241(d); 5 U.S.C. Sect. 552a; 44 U.S.C. Sect. 3501 et seq.
